# Editorial: Insights into molecular and cellular mechanisms of chronic pain and neuroinflammation

**DOI:** 10.3389/fnmol.2025.1679721

**Published:** 2025-08-29

**Authors:** Silke Neumann, Amit Sharma, Jarek Maciaczyk

**Affiliations:** ^1^Department of Pathology, Dunedin School of Medicine, Dunedin, New Zealand; ^2^Department of Integrated Oncology, Center for Integrated Oncology (CIO), University Hospital of Bonn, Bonn, Germany; ^3^Department of Stereotactic and Functional Neurosurgery, University Hospital of Bonn, Bonn, Germany; ^4^Department of Surgical Sciences, Dunedin School of Medicine, Dunedin, New Zealand

**Keywords:** neuroimmunity, neuroimmune crosstalk, chronic pain, signaling pathways, peripheral immunity

## Introduction

Chronic pain is an unresolved health issue affecting approximately a quarter of adults worldwide (Zimmer et al., 2022; Blyth and Huckel Schneider, 2018). Treatment of chronic pain focuses on removing the underlying cause of pain and managing pain symptoms with analgesics. Side effects of analgesia are common and novel targets, and medications are urgently needed.

Accumulating evidence suggests that neuroinflammation plays a crucial role in the development of chronic pain (Scheuren and Calvo, 2024; Grace et al., 2014; Chavan et al., 2017). Neuroinflammation is triggered by activation of non-neuronal cells, such as microglia and astrocytes, leading to the secretion of proinflammatory molecules (Cook et al., 2018; Pinho-Ribeiro et al., 2017). In addition to causing central sensitisation and chronic pain, proinflammatory molecules also attract peripheral immune cells into the tissue, perpetuating inflammation. Perhaps fueled by the discovery of functional lymphatic vessels that allow immune cells to egress the central nervous system (CNS) (Louveau et al., 2015; Aspelund et al., 2015), the field of neuroimmunology is rapidly evolving and shedding light on neuro-immune interactions that drive disease pathology.

This Research Topic brings together studies exploring the intricate crosstalk between the nervous and immune systems in the pathogenesis of chronic pain, neurological diseases, ageing and neurodegeneration. Spanning molecular, cellular, and translational research, these contributions highlight novel mechanisms and promising therapeutic targets for patients affected by neuroinflammation-driven conditions.

## Molecular and cellular mechanisms of neuroimmune signalling in pain

A central theme emerging across several studies is the critical role of neuroimmune signalling in shaping pain sensitivity and chronicity in the periphery and the CNS. For example, the Slack potassium channel (KCNT1) was identified as a key regulator of mechanical pain sensitivity through its action in dorsal root ganglion and spinal somatostatin-positive neurons, linking ion channel function to neuronal excitability and pain modulation (Liu et al.). Similarly, the trafficking and localisation of peripheral voltage-gated sodium channel NaV1.7, a validated target in human pain, were explored to dissect how intracellular transport mechanisms influence channel availability and function in sensory axons (Tyagi et al.).

Inflammatory mediators such as interleukin-6 (IL-6) and chemokines like CXCL1 further illustrate the dynamic neuroimmune milieu driving pain. The role of IL-6 in facilitating descending spinal sensitisation via JAK2/STAT3 and ERK signalling cascades exemplifies how supraspinal cytokine signalling propagates nociceptive hypersensitivity (Yang et al.). Parallelly, the upregulation of CXCL1 in the dorsal root ganglion and its contribution to hyperalgesic priming elucidate chemokine-driven neuroinflammation as an important mechanism in the transition from acute to chronic pain (Du et al.). Microglia, resident CNS macrophages, emerge as critical modulators of pain states, with pharmacological interventions shifting microglial phenotype from proinflammatory to neuroprotective via cannabinoid receptor 2 (CB2R) activation. This phenotypic plasticity is accompanied by changes in purinergic receptor P2X7 expression, linking microglial signalling pathways to analgesic outcomes (Zhou et al.). Inhibiting glial activity in the insular cortex also reduced chronic pain behaviours, highlighting the contribution of central glial-neuronal interactions in pain maintenance (Choi et al.).

Epigenetic regulation adds another layer of complexity. Studies uncovering the role of protein arginine methyltransferase 1 (PRMT1) in the anterior cingulate cortex (ACC) reveal how histone modification enzymes modulate pain hypersensitivity through fragile X mental retardation protein (FMRP) (Wu et al.). Additionally, a comprehensive review of miRNAs, including exosomal miRNAs, highlighted their emerging role in modulating neuropathic pain through regulation of neuroinflammation, nerve regeneration, and ion channel expression (Zhao et al.). Likewise, the reversible epigenetic silencing of cholinergic gene expression in basal forebrain neurons by REST and G9a histone methyltransferase, downstream of TLR4 activation, highlights inflammation-induced neuronal plasticity with implications for cognitive deficits and pain (Crews and Vetreno).

## Neuroimmune contributions to neurodegeneration and pain-related mood disorders

Neuroimmune pathways also play a central role in neurodegenerative processes and psychiatric co-morbidities. The well-documented link between chronic pain and depression is further elucidated through studies identifying a novel long non-coding RNA (lncRNA-84277) in the ACC that regulates SIRT1 expression via a competing endogenous RNA mechanism. This molecular axis modulates depression-like behaviours in chronic pain models, suggesting that targeting lncRNA-mediated epigenetic regulation could mitigate chronic pain (Jiao et al.).

A novel non-canonical inflammasome complex involving NLRP1 and caspase-8 was characterised in aged mouse cortex, underscoring inflammasome activation as a driver of CNS inflammaging. Therapeutic neutralisation of ASC, an adaptor protein in inflammasomes, attenuated neuroinflammation, revealing potential intervention points in age-related neurodegeneration (Cyr et al.).

## Clinical and translational insights

The clinical translation of mechanistic insights is a critical focus of this Topic. Pharmacological targeting of thrombin-PAR1 signalling in trigeminal neuralgia demonstrates that modulating local coagulation and blood-nerve barrier integrity relieved pain, highlighting the therapeutic promise of vascular-immune crosstalk modulation (Zhou et al.).

Novel opioid receptor agonists like NFEPP are designed to become active only under acidic conditions, which are typical of injured tissues. This allows them to selectively inhibit voltage-dependent calcium channels in sensory neurons while minimising side effects in the brain (Celik et al.).

Systemic immune profiling in musculoskeletal pain conditions, such as non-specific neck pain and cervical radiculopathy, showed increased levels of pro-inflammatory markers in the blood (Schipholt et al.). These immune changes were linked with factors like pain intensity, psychological stress, and lifestyle variables, including smoking and adiposity.

Moreover, the COVID-19 pandemic has spurred new investigations into viral protein-induced neuroimmune activation. SARS-CoV-2 envelope and spike proteins were shown to activate nociceptive signalling via TLR2/4-MyD88 pathways in sensory neurons, providing mechanistic insight into the somatosensory disturbances experienced by COVID-19 patients (Su et al.).

A bibliometric analysis of microglia-related neuropathic pain research reveals China's rising scientific output alongside the United States' ongoing influence. Emerging research trends include sexual dimorphism, oxidative stress, and stem cell therapies, pointing to future directions that warrant attention (Zhang et al.).

## Concluding perspectives and future directions

Taken together, the studies in this Research Topic highlight the multifaceted neuroimmune mechanisms underpinning pain and neurodegenerative disorders. The integration of molecular signalling, epigenetic regulation, glial biology, and systemic immune profiling offers a framework to identify novel biomarkers and therapeutic targets. Future research will continue to elucidate sex-specific differences, the bidirectional communication between neurons and immune cells, and the translational potential of emerging targets such as lncRNAs, inflammasomes, and purinergic receptors.

Harnessing these insights promises to accelerate the development of safer, more effective interventions that address both sensory and affective components of pain, as well as the cognitive and neuropsychiatric sequelae of neurodegeneration. This multidisciplinary endeavour will be vital to improving patient outcomes and alleviating the global burden of these debilitating conditions.
